# Infectivity and molecular epidemiology of Peste des Petits ruminants virus in slaughtered goats at the local meat market of Mymensingh division, Bangladesh

**DOI:** 10.5455/javar.2024.k815

**Published:** 2024-09-29

**Authors:** Sajeda Sultana, Munmun Pervin, Nazneen Sultana, Muhammad Tofazzal Hossain, RMd. afiqul Islam, Mohammad Abu Hadi Noor Ali Khan

**Affiliations:** 1 Department of Pathology, Sher-e-Bangla Agricultural University, Dhaka, Bangladesh; 2 Department of Pathology, Bangladesh Agricultural University, Mymensingh, Bangladesh; 3 Department of Microbiology and Hygiene, Bangladesh Agricultural University, Mymensingh, Bangladesh; 4 Bangladesh Agricultural Research Council, Dhaka, Bangladesh

**Keywords:** Goats, PPRV, phylogenetic analysis, RT-PCR protocols, slaughterhouse

## Abstract

**Objective:**

The purpose of this study was to characterize the circulating Peste des Petits Ruminants virus (PPRV) from slaughtered goats and conduct a phylogenetic analysis of the N gene of PPRV.

**Materials and Methods:**

A total of 196 slaughtered goats were investigated at the marketplaces of Mymensingh division from January 2019 to March 2021. Lungs, spleen, and mesenteric lymph nodes were collected for histology and molecular study. In-house developed Reverse-Transcriptase-Polymerase Chain Reaction (RT-PCR) protocols were carried out using designed primer sets (PPRV NF-gctctgtgattgcggctgagc and PPRV NR-cctggtcctccagaatcttggcc). The CLC sequence viewer was used for phylogenetic analysis.

**Results:**

Grossly pneumonic lungs, shrinkage spleen, and enlarged mesenteric lymph nodes with hemorrhages were recorded. Both intracytoplasmic and intranuclear inclusion bodies were seen in lymphocytes of the mesenteric lymph node, spleen, and lungs. PPRV was detected in 37 goats (18.9%) by RT-PCR test. The 402-bp amplicon was generated in PPRV-positive cases. The phylogenetic analysis showed that the studied PPRV isolates of the Mymensingh division belonged to lineage IV.

**Conclusion:**

The prevalence of PPR was 18.9% in slaughtered goats at marketplaces in the Mymensingh division. Slaughterhouses may be a source of PPRV, and it can be horizontally transmitted from the meat market to the farm. Restricting sick animal movement within the country, mass PPR vaccination campaigns, increased awareness, and improved biosecurity in the meat market may lessen the incidence of PPR in goats.

## Introduction

In Bangladesh, the number of goats was estimated to be 26.604 million in 2020–2021 [1]. Bangladesh’s most popular meat is mutton, which tastes better than beef, lamb, or chicken but costs more. Small ruminants served as a vital source of revenue and protein for household economics. Among infectious diseases, Peste des Petits ruminants (PPR, goat plague) is endemic and poses a danger to the livelihood of small ruminants in Bangladesh. PPR is an acute or subacute transboundary viral disease that is extremely contagious and economically significant, limiting sheep and goat production across Africa and Asia [2]. Although the actual annual losses in Bangladesh from PPR remain unknown, estimates suggest they surpass US$25 million [3]. The PPR virus (PPRV), which belongs to the Paramyxoviridae family and Morbillivirus genus, is the causative agent of PPR [4]. Considering the partial sequences of the N or F genes, the PPRV can be divided into four distinct lineages (I, II, III, and IV) based on its single serotype. Africa has recently detected all four lineages, while only lineage IV is circulating in Asia [5]. Multiple districts of Bangladesh frequently observe PPRV outbreaks, reporting a significantly elevated risk throughout the monsoon and winter seasons [6].

PPR-infected animals shed large amounts of the virus PPRV through their nasal and ocular secretions, saliva, and feces. Outside of the host, the PPRV is fragile and easily destroyed by heat and sunshine [7]. In addition, the disease can spread between animals by the intake of contaminated feed and water [3]. It is a common practice among poor goat owners that if the goats had been sick, they may have been sold at the neighborhood market. The infected goats were sometimes slaughtered at the meat market; this source could have transmitted PPR from slaughtered goats to farmer’s sheds. On the other hand, butchers collected goats from the village or local market. These goats were kept in a common shelter for a short period until slaughter.

Typically, animal transportation occurs through networks in the supply chain (herds-local-small and big markets to the central market). Bangladesh slaughters over 150 lakh goats annually, with 40% of them sacrificed for religious purposes during the “Eid-ul-Adha” holiday [8]. The extensive movement of cattle, sheep, and goats in the markets around the Eid festival (Eid-ul-Azha) facilitates the spread of PPRV along the supply chain. People primarily move animals through walk-by roads, boats (rivers), and transport networks. Infected animals shed PPRV when moving on the road, river, or in transport vehicles, allowing the virus to spread from one location to another [8]. Additionally, Bangladesh is already exporting modest amounts of mutton to a few nations, although the prevalence of PPR hinders larger-scale exports.

Following the eradication of rinderpest, the Food and Agriculture Organization and the World Organisation for Animal Health have chosen PPRV as the upcoming target for elimination by 2030 [9]. Knowledge of disease hotspots and risk factors would help policymakers in Bangladesh to develop effective control strategies [10]. It is essential to investigate the variety of PPRV strains in circulation to evaluate the efficacy and utility of accessible vaccines in Bangladesh [11]. All biocarriers and transporters need to restrict access to the hotspot that served as a source of PPRV. We need to effectively monitor PPR at either clinical or subclinical levels in slaughtered goats, using pathology, RT-PCR detection of PPRV, and PPRV genomics, to quickly assess PPRV infectivity and food safety levels. To select an effective vaccine and implement a vaccination campaign to eliminate PPR, we need to explore the genomics of PPRV from slaughtered goats. Recently, a couple of studies [9,11,12] examined and detected PPR infectivity in field and veterinary hospital cases by using PCR and sequencing; where the N, F, and H genes of the PPR virus were used in phylogeny. However, there is a lack of relevant data on PPRV isolation from slaughtered goats in the Bangladeshi meat market. This is the first study on laboratory confirmation and genetic characterization of PPRV in slaughtered goats at marketplaces in Bangladesh. Therefore, we designed this research to examine the pathology of the lungs, spleen, and lymph nodes in suspected goats. We developed an efficient RT-PCR protocol in-house to identify PPRV and carry out a phylogenetic analysis of the circulating PPRV in slaughtered goats from market sources.

## Methods and Materials

### Ethical approval

Bangladesh Agricultural University Research System (BAURES) has its own ethical standards committee to approve the research activity (BAURES/ESRC/VET/07-1/Jan 20, 2019).

### Sample size estimation

We used Cochran’s formula, a simple random sampling procedure, to select the required number of study animals. We checked a total of 196 slaughtered goats for PPR. We estimated the sample size using a 15% prevalence of PPR in goats [13], a 5% acceptable sample error, and a 95% confidence interval.

Where, *n = *required sample size,

Z = statistic for level of confidence,

P = Current prevalence of PPR in goats [13],

Q = (1-P),

e = Acceptable standard error.

### Collection of samples for histopathology and molecular study

We adopted strict biosafety and security measures during the collection, shipment, and processing of the samples. We investigated a total of 196 slaughtered goats in Mymensingh, Sherpur, Jamalpur, and Netrokona districts, Mymensingh division, from January 2019 to March 2021. We collected samples from 49 slaughtered goats in each district. We selected and examined the lungs, spleen, and mesenteric lymph nodes of slaughtered goats, which are the predilection sites of PPRV.

Following slaughter, we examined the lungs, spleen, and lymph nodes with the naked eye. We collected a portion of each goat’s lungs, spleen, and lymph nodes in 10% neutral buffered formalin and sterile cryotubes for histological analysis and molecular study, respectively. Then, hematoxylin and eosin staining were used for histopathological study [14] and observed at low (10x) and high (40x and 100x) power magnifications (Cell Bioscience, Alphaimager HP, California, USA).

### RT-PCR detection of PPRV

Approximately 100 mg of lung tissue, 60 mg of spleen, and lymph nodes were separately triturated (Qiagen Tissue Lyser-II, USA) from each goat. The Eppendorf tube containing tissue homogenates was centrifuged at 5,000 rpm for 5 min [15]. PPR viral Ribonucleic acid (RNA) was extracted from tissue fluid using a commercially available RNA extraction kit (SV total RNA isolation system, Promega). In our earlier investigation, we created oligonucleotide primers that were based on a portion of the N gene of PPRV [15] and used to detect PPRV from the slaughtered goats. The forward PPRV N gene forward primer (5’-gctctgtgattgcggctgagc-3’) and reverse PPRVNR (5’-cctggtcctccagaatcttggcc-3’) primers [15] were obtained commercially from Macrogen, Korea.

The one-step RT-PCR kit (New England, Biolab Inc., USA) was utilized for the RT-PCR procedure (Proplex gradient PCR, USA). The 50 μl reaction volume master mix consists of a 25 μl *Taq* reaction mix (2X), 2 μl enzyme mix (25X), 2 μl forward and reverse primers (10 pmol/μl each), 17 μl nuclease-free water, and 2 μl template RNA. We followed our developed RT-PCR protocol to amplify a 402-bp fragment of the N gene of PPRV [15]. The 40 cycles of reaction consist of reverse transcription at 48°C for 20 min, first denaturation for 1 min at 94°C, denaturation at 94°C for 15 sec, annealing at 55°C for 30, elongation at 68°C for 1 min, and final extension at 68°C for 5 min. 1.5% agarose gel containing ethidium bromide (0.5 g/ml) was used for electrophoresis (WSE-1710 Submerge-Mini2322100, China), and images were documented (Alpha Imager, USA). RNA from healthy lung tissues was as a negative control, and a 100-bp Dioxy ribonucleic acid (DNA) ladder (TackIT, Invitrogen, USA) was used for amplicon size estimation.

### N gene sequence analysis

We sequenced the positive cDNAs (Macrogen Inc., Korea) and initially identified them using the online Basic Local Alignment Search Tool (BLAST). We downloaded the relevant sequence data from the National Centre for Biotechnology Information (NCBI), which included isolates collected from various countries and years. We submitted the N sequences of PPRV to GenBank to obtain the accession number. CLC Sequence Viewer 8 was used to perform phylogenetic analysis by performing multiple sequence alignments using UPGMA, Kimura 80, and Maximum Likelihood (MJ) tests [16]. The reliability of the phylogenetic tree was examined using 100 Bootstrap value replicates.

### Data analysis

The statistical method SPSS-20 was used for the analysis of data.

## Results

### Gross pathology

**Figure 1.**
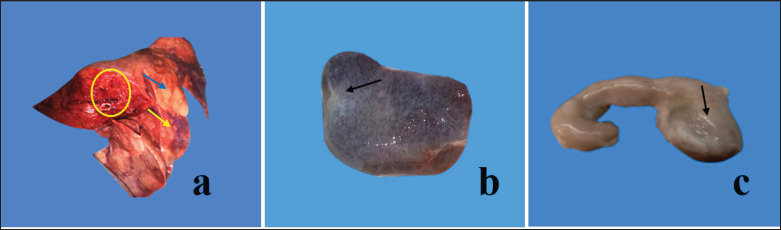
The lungs of slaughtered goats suspected to infect with PPR showed congestion and consolidation with foamy exudation on the cut surface (a, circle). Gray hepatized (a, blue arrow) and widespread congestion (a, yellow arrow) were seen in the lungs of goats. The fibrinous covering with shrinkage spleen (b, arrow) was seen in PPR-infected cases. Enlarged/ edematous/hemorrhagic mesenteric lymph nodes were seen in slaughtered goats (c, arrow).

The lungs, spleen, and mesenteric lymph nodes obtained from goats of several slaughterhouses of four districts of Mymensingh division (*n = *196) revealed pneumonic lungs in 80 goats (Fig. 1a). Fibrinous covering with shrinkage spleen indicating atrophy was observed in 33 goats (Fig. 1b), and hemorrhagic/edematous and enlarged mesenteric lymph nodes were seen in 92 goats (Fig. 1c).

### Histopathology

**Figure 2.**
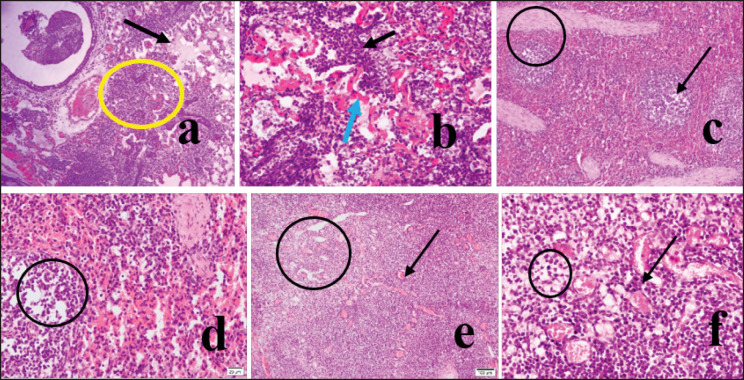
In H&E satin, the lung tissues showed hemorrhages with massive infiltration of reactive cells (10X, a, circle), mainly lymphocytes and neutrophils (40X, b, black arrow), and hemorrhages from the alveolar-capillary (40X, b, blue arrow). The lung’s alveoli were sunk in exudate and appeared light pink (10X, a, arrow). There was lymphoid depletion (10X, c, arrow and 40X, d, circle) and enlargement of trabeculae (10X, c, circle) in the infected spleen. Lymphoid depletion was also observed in mesenteric lymph nodes (4X, e, circle and 40X, circle). There were congestion and hemorrhages in mesenteric lymph nodes (40X, b, arrow).

**Figure 3.**
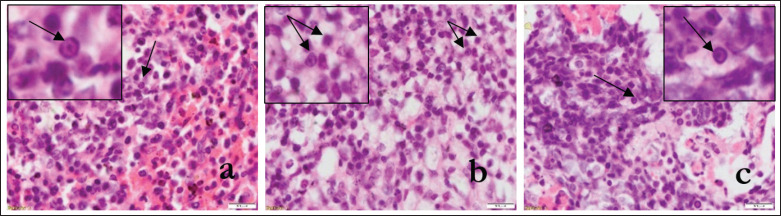
In H&E satin, intranuclear inclusion body in lymphocytes of the spleen (40X, an arrow, and 100X, inset, arrow) was seen. Both intracytoplasmic and intranuclear inclusion bodies in lymphocytes of mesenteric lymph node (40X, b, arrow and 100X, inset, arrow) and intranuclear inclusion body in lymphocytes of the lungs (40X, c, arrow and 100X, inset, arrow) was seen.

The lungs of PPRV-infected goats appeared congested, and the alveoli showed massive infiltration of reactive cells (Fig. 2a, circle). There were hemorrhages in the lungs, and hemorrhagic blood was seen to escape into the lung alveoli (Fig. 2b, blue arrow). Widespread infiltration of inflammatory cells, mainly lymphocytes and neutrophils, was seen along with hemorrhages (Fig. 2b, black arrow). The lung’s alveoli were sunk in fibrinous exudate, which appeared light pink (Fig. 2a, arrow). The bronchioles were inflamed, containing reactive cells with hemorrhages. There was lymphoid depletion (Fig. 2c, arrow and 2d, circle) and trabecular enlargement (Fig. 2c, circle) in the spleen. Lymphoid depletion was observed in mesenteric lymph nodes (Fig. 2e and f, circle) with congestion and hemorrhages (Fig. 2f, arrow). Intranuclear inclusion bodies were seen in lymphocytes of the spleen (Fig. 3a, arrow and 100X, inset, arrow). Both intracytoplasmic and intranuclear inclusion bodies in lymphocytes of the mesenteric lymph node (Fig. 3b, arrow and 100X, inset, arrow) and intranuclear inclusion bodies in lymphocytes of the lungs (Fig. 3c, arrow and 100X, inset, arrow) were seen.

**Figure 4.**
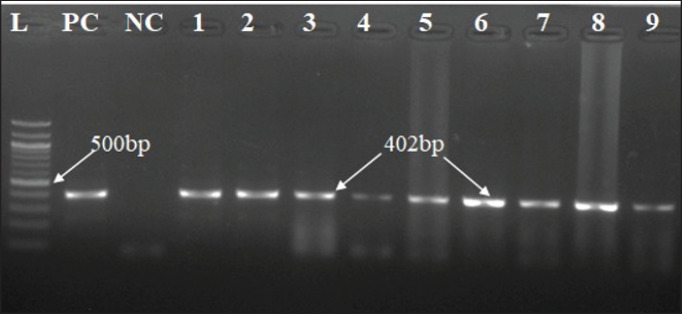
Amplification of the N gene of the PPR virus using RT-PCR with the viral RNA extracted from the lungs, spleen, and mesenteric lymph nodes of infected goats. In positive cases, a 402-bp amplicon was generated. Test samples are in lanes 1 through 9, the 100 bp ladder is in lane L, and the positive and negative controls are in PC and NC, respectively.

**Figure 5.**
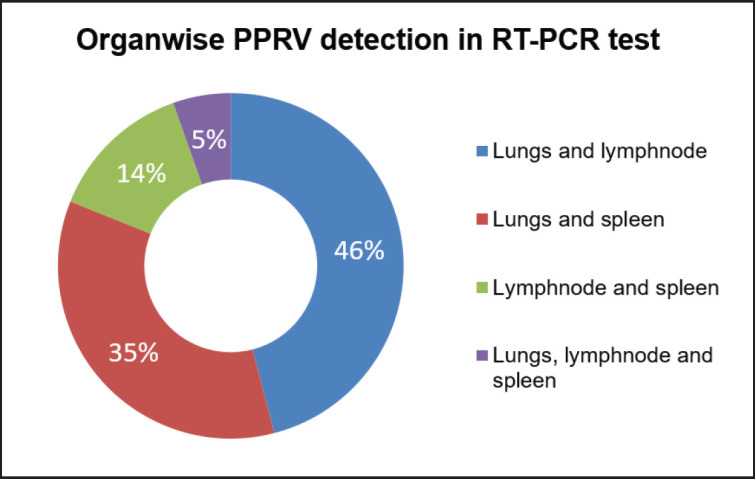
Representation of the infectivity of slaughtered goats (*n = *37, 18.9%) in visceral organs due to PPRV as detected by RT-PCR. The highest number of positive yields was seen in both lungs and lymph nodes (46% cases) followed by both lungs and spleen (35% cases), both lymph nodes and spleen (14% cases), and least in all organs like lungs, lymph nodes, and spleen (5% cases).

**Figure 6.**
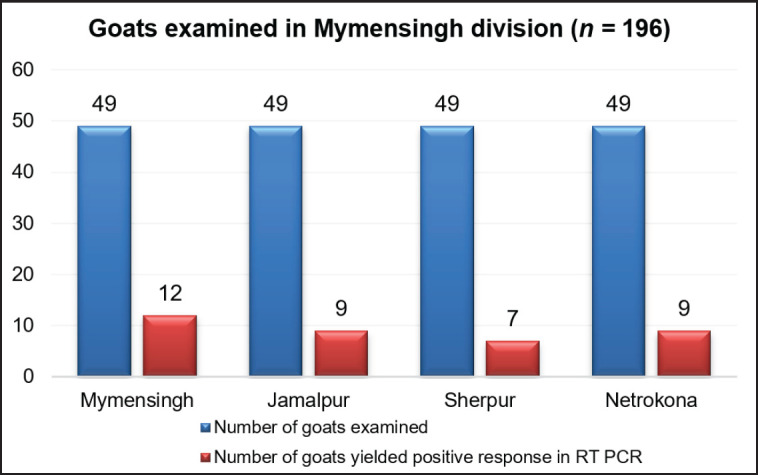
Detection of district-wise PPRV infectivity in slaughtered goats. Out of 196 slaughtered goats examined in four districts of Mymensingh division, 37 (18.9%) yield PPR viral (N gene) specific 402bp amplicon in RT-PCR. Results showed PPRV infectivity in 12 cases (33%), 9 cases (24%), 7 cases (19)% and 9 cases (24%) at Mymensingh, Jamalpur, Sherpur, and Netrokona districts, respectively.

**Figure 7.**
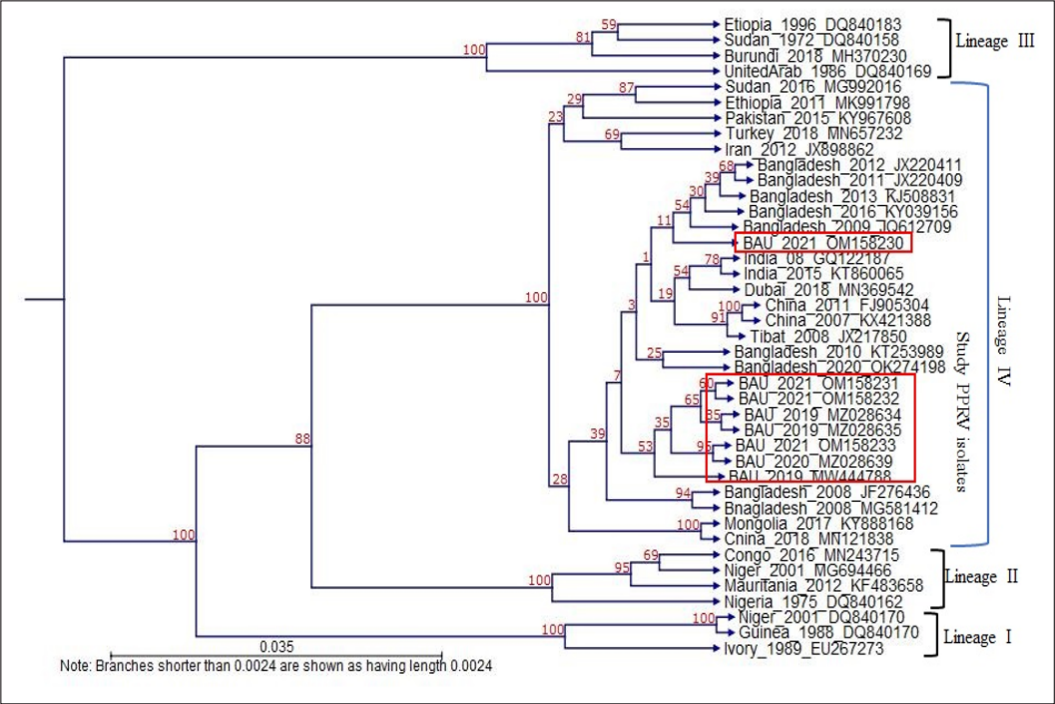
The phylogenetic relationship was determined by analyzing the partial N gene sequences of PPRV isolates in this study and in comparison to other typical PPRVs from Lineages I-IV. The N gene of eight PPRV isolates from the Mymensingh division formed a separate subcluster with lineage IV isolates from Bangladesh and Asia.

### Molecular identification of PPRV 

RNA extracted from the lungs, spleen, and mesenteric lymph nodes of 196 slaughtered goats and used in RT-PCR amplification amplified a 402-bp fragment of the N gene of PPRV in 37 goats (Fig. 4). Any organ positive for PPRV was considered PPR viral infectivity. PPR-positive viral RNA from the lymph nodes and lungs of 17 goats (46%), the lungs and spleen of 13 goats (35%), the lymph nodes and spleen of 5 goats (14%), and RNA from all samples such as the lungs, lymph nodes, and spleen of 2 goats (5%) yielded a PPRV-positive amplicon in the RT-PCR test (Fig. 5). Organwise PPRV distribution was highest in lungs (32 cases), followed by lymph nodes (24 cases) and spleen (20 cases). In this study, PPRV infectivity appeared highest in 12 cases (33%) of Mymensingh district, 9 cases in Jamalpur district (24%), 7 cases in the Sherpur district (19%), and 9 cases in the Netrokona district (24%) of Mymensingh division (Fig. 6).

### Phylogenetic and nucleotide sequence analysis 

Eight PPRV (08) positive cases were sequenced, analyzed, and submitted to GenBank. Four partial N gene-specific sequences of PPRV from Mymensingh district, 02 from Netrokona district, 01 from Jamalpur district, and 01 from Sherpur districts got GenBank accession (MZ028634, MZ028635, MW444788, MZ028639, OM158230, OM158231, OM158232, and OM158233).

The nucleotide sequence of 8 PPRV isolates from the Mymensingh division that were analyzed in BLAST exhibited the highest nucleotide homology with the Bangladesh/2016/OK274194 isolates (Mymensingh PPRV isolates: 98.24%–99.74%, Netrokona PPRV isolates: 99.19%–99.74%, Jamalpur PPRV isolate: 99.20% and Sherpur isolate: 98.38%), followed by the China/2007/KX421388 (96.73%–98.15%), India/2016/KX033350 (96.48%–97.87%), Dubai/2018/MN369542 (95.42%–96.83%), and Israel/2014/OL310704 (95.15%–96.56%) isolates.

Eight PPRV isolates were investigated, and the N gene sequences were used in phylogenetic analysis in contrast to those of other representative PPRV isolates from Lineages I to IV. The study isolates nucleotides had homology with other Asian and African isolates such as India, China, Dubai, Iran, Pakistan, Turkey, Sudan, and Mongolia belonging to lineage IV (Fig. 7). The 8 studied N genes of PPRV isolates revealed that the study isolates made a separate cluster and also segregated into separate subclusters among the study isolates (Fig. 7). The cluster of studied isolates was very close to other previous Bangladeshi PPRV isolates. This study revealed that the N gene of PPRV evolves continuously.

## Discussion

Four steps are involved in the global objective of eradicating PPR by 2030: evaluation, control, eradication, and post-eradication follow-up [17]. To effectively control PPR, it is critical to have a thorough understanding of PPR disease epidemiology. Additionally, it is imperative to provide robust backing for diagnostic techniques and ensure the timely administration of vaccinations to the susceptible population. The national control program for PPR in India is strongly recommended due to the accessibility of attenuated cell culture vaccines as well as different diagnostic procedures/kits for the rapid diagnosis of PPR [18]. Vaccination and recovery from PPR typically induce a lifelong immune response; however, revaccination is necessary every 3 years to maintain a protective level of immunity. To halt the virus’s transmission, widespread immunization programs need to achieve high herd immunity of 70%–80% [19]. Foot-and-mouth disease (FMD) has 7 serotypes, and vaccination against 1 serotype does not save against infection with the other serotypes. PPR is easier to control than FMD because of PPR viral antigenic strength, a single serotype, and the production of a lifelong immune response following vaccination.

A National strategy plan (NSP) has been developed by Bangladesh in the framework of the international initiative to control and eradicate PPR. The NSP is now seeking approval. In certain districts where goat-rearing is prevalent, namely Jashore, Chuadanga, Meherpur, and Kustia, notable advancements have been made in the PPR control program. These advancements encompass targeted mass vaccination, heightened farmer awareness, and active participation in the program. Nevertheless, it is important to note that vaccination coverage remains unattained in other districts where goat-rearing is practiced. The PPR vaccine is generally procured by diligent farmers from sub-district and district veterinary hospitals, where it is available in limited quantities. Nevertheless, occasional vaccination campaigns are meant to raise farmers’ awareness of the disease [8]. In some goat-rearing zones, where animal migration is especially high, a mass immunization campaign has proven to be protective [20].

Animals are commonly transported through supply chain networks, which involve the movement of herds from local minor markets to larger central markets. Animal movement is tough to manage during festivals and all year. Goats were then collected from the local market for slaughter purposes. All goats were transported to the city using common vehicles and kept in a common shelter for a short period until slaughter. PPR transmission may occur during this transportation. These goats were randomly slaughtered according to market demand. We attempted to find out the prevalence of PPR in slaughtered goats using PCR techniques in the laboratory. For that reason, 196 slaughtered goat samples (lungs, spleen, prescapular, and mesenteric lymph nodes) were collected from different slaughterhouses of the Mymensingh division. During sample collection, emphasis was given to slaughtered goats with congested lungs, enlarged lymph nodes, and fibrinous spleen. Previously, a study examined 24 slaughtered goats in Mymensingh. PPRV was detected in the lymph nodes of 2 cases (8.3%) by RT-PCR [21]. This study examined extensively different organs (lungs, lymph nodes, and spleen) of slaughtered goats and detected PPRV at a higher rate (18.9%, *n = *37). The PPRV infectivity rate appeared higher, which could be due to the selection of different organs at slaughter.

Histopathologically, lung alveoli were distended, ruptured, and contained fibrinous inflammation. There was widespread infiltration of inflammatory cells, mainly lymphocytes and neutrophils, in the lungs. Lymphoid depletion is a common phenomenon in PPR-infected goats [3,18]. In this study, lymphocytic depletion was seen in lymph nodes and spleen. The histopathological observation of lymphoid organs and lymphocyte depletion supported infectivity due to PPRV. Pathognomonic lesions, as seen in PPRV-infected goats, are the development of intracytoplasmic and intranuclear inclusion bodies in lungs, spleen, and lymph node cells and were detected by H&E staining. Histopathological findings predominantly observed were widespread hemorrhages and congestions in the lungs, spleen, and lymph nodes with widespread lymphoid depletion in lymphoid organs; these changes are in accordance with the previous findings of PPRV infectivity [3,18,21].

Genetic studies with the PPRV indicated that the PPR virus contains RNA genomes and thus continuously evolves. The PPR virus changes its genome constantly during the development and propagation cycles. In the Middle East and Asia, PPRV strains primarily belong to lineage IV, but lineages III and IV were recorded in Saudi Arabia [22]. Interestingly, viral RNA is generally limited to lymph nodes, while viral DNA is distributed to a variety of tissues [23]. Sandwich ELISA revealed that PPR viral antigen was present in 75% of intestinal mucosa, 66.67% of blood samples, 62.5% of lymph nodes, 60% of samples from a combination of the heart, kidney, and liver, 57.84% of lung, 50% of spleen, and 40% of nasal swabs [22]. In this investigation, RNA was taken from the lungs, lymph nodes, and spleen of each slaughtered goat and used in RT-PCR, 37 goats tested positive for PPRV. RNA from the lungs and lymph nodes of 17 goats used in RT-PCR yielded the highest (46%) amplification of the 402-bp fragment. However, the PPRV-specific amplification in RT-PCR was 35% (*n = *13) with the viral RNA collected from the lungs and spleen. The amplification potential in RT-PCR was lower (14%) with the viral RNA collected from lymph nodes and spleens (*n = *5). The amplification potential in RT-PCR was lowest (05%) with the viral RNA collected from all organs like lungs, lymph nodes, and spleen (*n = *2). The variation of amplification potentials may be because the tissue RNAse may have contaminated lungs, lymph nodes, or spleen during collection or viral RNA extraction procedure, and the variable level of loads of viral RNA in tissues collected or RNA may have disappeared in PPRV-infected goats during subclinical/longer/chronic period of infectivity. This study found that RNA extraction from the lungs and spleen was much easier than lymph nodes. Because lymph nodes were sticky, and most of the time they blocked the RNA filter during the extraction procedure. However, it needs investigation at a larger scale to avoid discrimination and develop a more sensitive procedure.

PPRV isolates from Bangladesh and India have molecular evidence of transboundary transmission [9]. Previous studies reported the PPRV endemicity in goats in Mymensingh and Netrokona [11,12,24]. Bangladeshi isolates were extremely close to China/Tibet 2007 isolates, with 94.5%–98.8% homology [12]. In this study, BLAST analysis of the N gene fragment of studied PPRV isolates (*n = *8) showed that the studied isolates were closed to former Bangladeshi isolate/2016/OK274194 (98.24%–99.74%), followed by China/2007/KX421388 (96.73%–98.15%), India/2016/KX033350 (96.48%–97.87%), Dubai/2018/MN369542 (95.42%–96.83%), and Israel/2014/OL310704 (95.15%–96.56%), which is in line with previous investigation [11,12].

In this study, phylogenetic analysis based on N gene sequences of 8 PPRV isolates revealed that the studied isolates were clustered closely with the isolates of China, India, Dubai, Iran, Pakistan, and other Asian countries belonging to lineage IV; this is an expected observation [9]. This study revealed that PPR viruses were prevalent in slaughtered goats during the winter season, which is inconsistent with the previous study [21]. Samples collected from the goats of the slaughterhouses of Mymensingh division may have been either acutely infected (slaughtered before death), subclinically infected, or recovered following infection with PPRV. Results of gross and microscopic investigation of visceral organs of slaughtered goats showed hemorrhagic lymph nodes in 92 cases, pneumonic lungs in 80 cases, and atrophied spleen in 33 cases, but RT-PCR amplification documented the presence of PPRV in 37 cases. RT-PCR is a more sensitive and rapid diagnostic tool than the pathological examination of lesions in the lungs, lymph nodes, and spleen. Out of 196 slaughtered goats examined in this study, 37 were confirmed to be infected with PPRV, indicating a large number of acutely, subacutely, chronically, or recovered goats following infection were slaughtered in various meat markets of Mymensingh division. Some other concurring infection with PPRV or some of the infection alone may be present in the slaughtered goats, including *Pasteurella multocida*,* Mycoplasma *spp*.*,* Streptococcus *spp*.*,* Staphylococcus *spp*.*,* Corynebacterium* spp., etc. It needs to test slaughtered samples for all the infectivity as cited above and needs to collect samples from all the 08 divisions of Bangladesh. If samples were collected during all the seasons and from all other divisions of Bangladesh, then the exact prevalence rate of PPRV in slaughtered goats would come out, which could bring critical ideas for controlling PPRV in Bangladesh.

## Conclusion

Small ruminant trade, particularly in meat markets where animals from different sources come into close contact with one another, enhanced the possibility of PPR viral transmission. The use of biosecurity in the meat market of Bangladesh is poor, and there is a lack of regulatory veterinary care. PPR is a contagious viral disease with high morbidity and mortality, and the infected goats lived for a few days before being sold in the meat market. To determine PPR viral infectivity in slaughtered goats, a confirmatory, sensitive diagnostic technique is required. PPR virus-specific RT-PCR protocol using designed primers was used in this investigation to detect a fragment of the N gene from slaughtered goats. The prevalence of PPR was 18.9% in slaughtered goats at marketplaces in the Mymensingh division. Phylogenetic analysis revealed that these PPR viruses belong to lineage IV. Slaughterhouses may be a source of PPRV, and it can be horizontally transmitted from the meat market to the farm. Restricting sick animal movement within the country, mass PPR vaccination campaigns, increased awareness, and improved biosecurity in the meat market may lessen the incidence of PPR in goats in Bangladesh. A vast number of slaughtered goat samples from seven other districts must be studied to establish future preventive and control strategies against PPR in small ruminants.

## List of Abbreviations

DNA, Dioxy ribonucleic acid; FAO, Food and Agricultural Organization; PPR, Peste des Petits Ruminants; PPRV, Peste des Petits Ruminants virus; PPRVNF, PPRV N gene forward primer; PPRVNR, PPRV N gene reverse primer; N gene, Nucleoprotein gene; RT PCR, Reverse transcriptase polymerase chain reaction; WOAH, World Organization of Animal Health; RNA, Ribonucleic acid; %, Percent; ºC, Degree celsius; μl, Microlitre; gm, Gram; bp, Base pair; cDNA, Complementary DNA
